# A Study Linking Axial Length, Corneal Curvature, and Eye Axis With Demographic Characteristics in the Emmetropic Eyes of Bangladeshi People

**DOI:** 10.7759/cureus.29925

**Published:** 2022-10-04

**Authors:** Maskura Benzir, Akhtari Afroze, Afroj Zahan, Rawshon Ara Naznin, Afsana Khanam, Sharmin A Sumi, Md. Ahsanul Haq, Halyna Lugova, Mainul Haque

**Affiliations:** 1 Anatomy, Thengamara Mohila Sabuj Sangha (TMSS) Medical College, Bogra, BGD; 2 Anatomy, Rajshahi Medical College, Rajshahi, BGD; 3 Anatomy, Gazi Medical College, Khulna, BGD; 4 Anatomy, Bangabandhu Sheikh Mujib Medical University (BSMMU), Dhaka, BGD; 5 Statistics, Infectious Diseases Division, International Centre for Diarrhoeal Disease Research, Bangladesh (icddr,b), Dhaka, BGD; 6 Humanitarian Assistance and Disaster Relief Research Centre and Community Medicine, Faculty of Medicine and Defence Health, Universiti Pertahanan Nasional Malaysia (National Defence University of Malaysia), Kuala Lumpur, MYS; 7 Pharmacology and Therapeutics, Universiti Pertahanan Nasional Malaysia (National Defence University of Malaysia), Kuala Lumpur, MYS

**Keywords:** optical parametric measurement, gender comparison, eye axis, bangladesh, descriptive cross-sectional analytical study, association, emmetropic eye, keratometry, corneal curvature, axial length

## Abstract

Background

Axial length (AL) and corneal curvature (CC) are one of the furthest critical parameters for optometry and oculoplastic surgery. These two variables are crucial in biometry for accurately measuring the power of the intraocular lens in cataract surgery. This research aimed to determine the association linking axial length and corneal curvature with demographic characteristics in emmetropic eyes of Bangladeshi people.

Methods

This descriptive cross-sectional research was carried out among 200 emmetropic eyes of Bangladeshi people attending the Department of Ophthalmology at Rajshahi Medical College, Bangladesh, with different eye conditions, between July 2017 and June 2018. Data was gathered by conducting person-to-person interviews, checking visual activity using the Snellen chart, and measuring corneal curvature using an auto-keratometer and axial eyeball length using A-scan ultrasonography.

Results

A total of 200 attendances were studied, 90 males and 110 females. All were emmetropic. The age range was 21-52 years, and the highest contributors were in the 21-30-year age group. The association between right axial length and right corneal curvature shows a negative relation among both sexes. It was -0.61 (β-coefficient (β-coff)), and highly significant in females at -0.89 (β-coff). Additionally, the association between left axial length and left corneal curvature shows a negative relation of -0.65 (β-coff), which was again highly significant in females at -0.87 (β-coff). Both were not significant in males. There was no significant association linking axial length and eye axis in both sexes. The multivariate regression model was used to assess the p-value, and the regression model was adjusted by age.

Conclusion

Optical parametric measurement is a noninvasive diagnostic and assessment tool that might help in the actual measurement of intraocular lens implantation in cataract surgery and may also provide supplementary information to the researcher domain.

## Introduction

Anatomy

The eye is the most important sense organ and organ of vision in humans. This is the peripheral organ of vision. The eyeball is situated in the orbit of the skull. Eyeballs are a cystic structure and almost spherical in shape [1]. The anterior and posterior diameter (axial length (AL)) is usually about 22-24 mm in the emmetropic eye [2]. The parallel rays of light from any image that enters the eye are focused on the retina by the refractive properties of the cornea and lens [3]. So, to achieve emmetropia, the axial length (AL) and corneal curvature (CC) have a definite relation. Emmetropic eyes’ visual acuity is 6/6 because the patient’s sitting or standing position is 6 m from the Snellen or E chart [4]. Usually, the axial length is the length from the corneal surface to an intervention peak in tune with the retinal pigment epithelium or Bruch’s membrane [5-7].

On the other hand, the cornea is the outer covering of the eyeball, which is transparent and avascular and protects the eye against different infections [8]. About 70% of refraction of the eye is contributed by the cornea, contributing about 40-44 D of refractive power [7]. Various refractive errors arise if these two variables are not appropriately coordinated. In other studies, refractive flaws are the second highest cause of blindness [8]. In Bangladesh, the prevalence of refractive error (RE) was 4.7% [9]. Around 1-2 billion people suffer from refractive error globally [10].

Embryology

Usually, most eye development occurs in the first 18 months with the progressive flattening of the cornea [11]. The cornea is developed from three sources: corneal epithelium derived from surface ectoderm, neural crest cell, and mesenchyme originating from mesoderm [12]. In normal eyes, they exceeded the progressive corneal flattening resulting from overall axial length changes. The normal physiology of axial length elongation starts during 3-6 months of life, progressively decreasing over the next two years [13] and completing the development within the first few years of life [14]. It has been reported that throughout the first year of ocular growth in humans, the cornea and lens lose power while axial length grows [15,16]. The corneal power remains the same after age 3 [17], with about 32 genetic markers related to corneal curvature and axial length development [18]. Usually, emmetropia arises between three and nine months of age [19]. Genetic influences and lifestyle are also responsible for the differences in corneal curvature measurement [20-24].

Clinical implication of emmetropic process

The human eye is planned to attain emmetropia in teens and preserve emmetropia with aging [25]. Many major eye diseases are related to corneal curvatures, such as keratoconus, myopia, and corneal astigmatism [26]. The quality of cataract surgery has advanced much in the last few decades [27]. Visual acuity after cataract surgery improved tremendously [28,29]. Additionally, patient side exception has been raised a lot in correcting refractive error after surgical intervention of cataract [30-32]. The importance of eyeball biometry is an absolute need for intraocular lens power calculations [30,33]. The axial length is related to myopic refractive error distressing, affecting a wide range of age groups [34,35]. The World Health Organization (WHO) reported that refractive defects of the eye are the primary starting point of visual disability and blindness globally [10,36,37].

Furthermore, over two-fifths of visual disablement are ascribed to refractive flaws [38]. Additionally, hyperopia and myopia increase the risk of all frequently occurring glaucoma [1,39]. Farsightedness (hyperopia) is a recurrent visual disorder wherein a patient can see far-off things distinctly, nonetheless close-by items, perhaps fuzzy. Myopia (near-sightedness/short-sightedness) is a kind of refractive defect. Myopic patients’ light focuses ahead of, in lieu of, the retina. It has been reported that myopia positively correlates with an upper pervasiveness of every single category of open-angle glaucoma and ocular hypertension. However, hyperopia was substantially related to a higher prevalence of primary angle-closure glaucoma [39-42]. In Bangladesh, the population aged 40-49 years had a higher significance of hyperopia than the relatively young people of 30-39 years [43].

This study intended to find the association between axial length and corneal curvature in the emmetropic eye of people in northern Bangladesh.

## Materials and methods

Study design

This was a cross-sectional research study.

Study population

The study populations were emmetropic patients at the Department of Ophthalmology, Rajshahi Medical College, Rajshahi, Bangladesh.

Study period

Data were collected between July 2017 and June 2018.

Sampling methods and sample size

A universal sampling method was adopted, and the sample size was 200.

Data collection techniques

A survey questionnaire was prepared to collect data. Non-probability sampling was used to select the sample population.

Ethical consideration

This study obtained Institutional Review Board (IRB) approval from Rajshahi Medical College, Rajshahi, Bangladesh (reference number: RMC/ERC/2017-2019/75) on December 3, 2017. Additionally, all research participants were adequately briefed about scientific publications. Consequently, written informed consent was obtained before any intervention was conducted.

Statistical analysis plan

The demographic characteristics of the study participants were calculated based on age and sex. Univariate or bivariate regression models were used to assess the association between outcomes (corneal curvature and eye axis) and predictor variables (axial length). The Student’s t-test was used to see the mean difference between male and female axial length and corneal curvature. A multivariate regression model was used to estimate the association between outcomes and predictor variables, and the model was adjusted by age. A p-value of <0.05 was considered significant. All data were analyzed using the Statistical Package for the Social Sciences (SPSS) for Windows version 20 (IBM SPSS Statistics, Armonk, NY, USA) and Stata/IC version 15 (StataCorp LLC, College Station, TX, USA).

## Results

**Figure 1:**
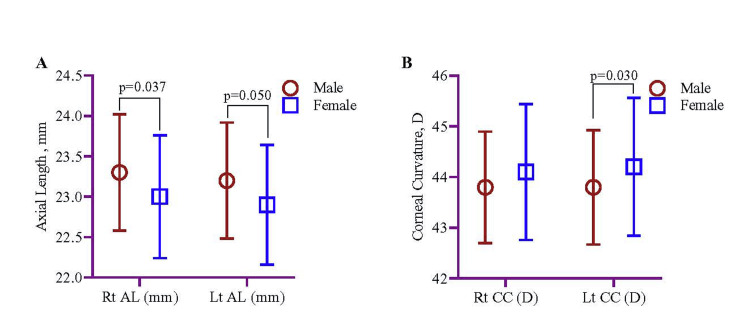
Distribution of Rt AL, Lt AL, Rt CC, and Lt CC stratified by sex. Data were presented as mean with SD, and the mean difference between males and females was estimated using the Student’s sample t-test. Rt AL: right eye axial length; Lt AL: left eye axial length; Rt CC: right eye corneal curvature; Lt CC: left eye corneal curvature; mm: millimeter; D: diopter; SD: standard deviation

The sociodemographic details of the study participants are depicted in Table 1. Among 200 study participants, 90 (45%) and 110 (55%) were male and female, respectively. Additionally, the age distribution of this study’s participants was described in Table 2. The right (p=0.037) and left (p=0.050) axial lengths of male participants were statistically significantly higher than those of female participants. Left corneal curvature was statistically significant (p=0.030) in females than in males (Table 3 and Figure 1).

**Table 1:** Distribution of the total number of participants and the total number of eyes according to sex.

	Number of cases	Number of eyes	% of cases	Age
Male	90	180	45	29.0±6.51
Female	110	220	55	29.6±6.27
Total	200	400	100	29.3±6.37

**Table 2:** Age distribution of the study participants (N=200). Groups were created for every 10 years of age stratification. SD: standard deviation

Age distribution	Overall	Male	Female	p-value
Age (mean±SD)	29.3±6.37	29.0±6.51	29.6±6.27	0.551
21-30	117 (58.5%)	55 (47%)	62 (53%)	0.467
31-40	75 (37.5%)	31 (41.3%)	44 (58.7%)	0.089
>41	8 (4%)	4 (50%)	4 (50%)	0.999

**Table 3:** Rt AL, Lt AL, Rt CC, and Lt CC stratified by sex. Rt AL: right eye axial length; Lt AL: left eye axial length; Rt CC: right eye corneal curvature; Lt CC: left eye corneal curvature; mm: millimeter; D: diopter

Parameters	Overall (N=200)	Male (n=90)	Female (n=110)	p-value
Rt AL (mm)	23.1±0.75	23.3±0.72	23.0±0.76	0.037
Lt AL (mm)	23.1±0.74	23.2±0.72	22.9±0.74	0.050
Rt CC (D)	44.0±1.24	43.8±1.10	44.1±1.34	0.168
Lt CC (D)	44.0±1.27	43.8±1.13	44.2±1.36	0.030

**Figure 2:**
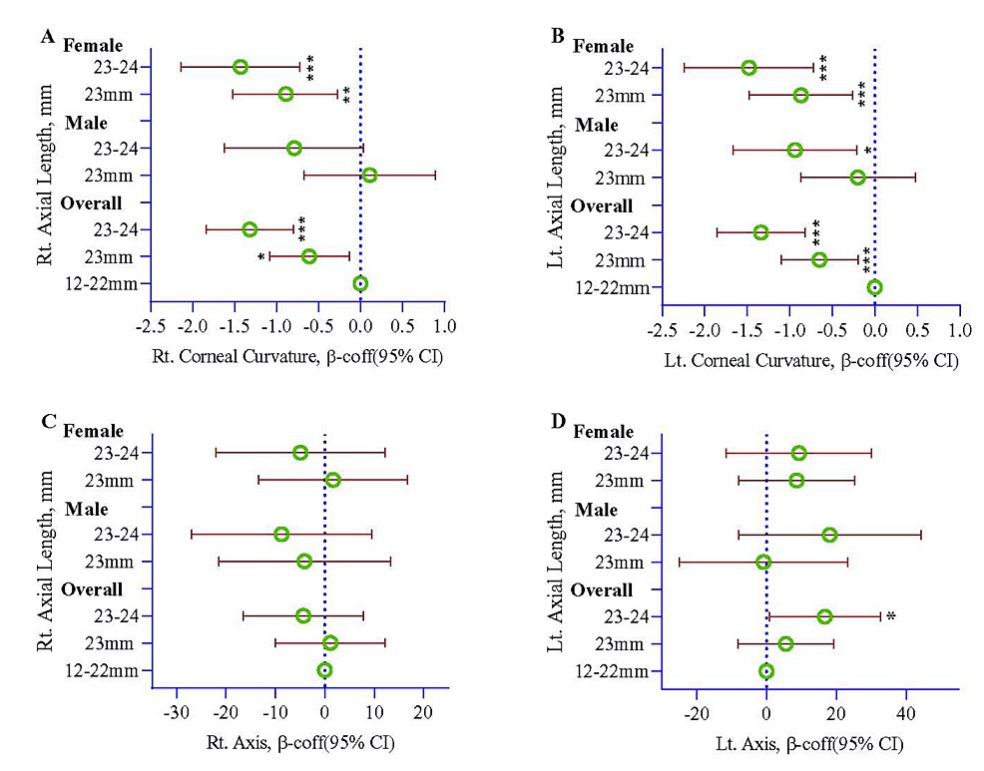
Association of right axial length with right corneal curvature (A), left axial length with left corneal curvature (B), right axial length with right axis (C), and left axial length with left axis (D) estimated using a multivariate regression model, and the model was stratified by sex. The regression model was adjusted by the age of the participants. mm: millimeter; D: diopter; Rt: right; Lt: left; β-coff: β-coefficient; 95% CI: 95% confidence interval

Rt AL at 23 mm decreased 0.61 mm of Rt CC compared to Rt AL at 21-22 mm (reference) (β-coefficient (β-coff)=-0.61; 95% confidence interval (95% CI)=-1.08, -0.13; p=0.013); the decline also remains significant for female participants (β-coff=-0.89; 95% CI=-1.52, -0.27; p=0.006), but not for male. The estimated Rt CC declined more when Rt AL was at 24-25 mm (β-coff=-1.32; 95% CI=-1.84, -0.80; p<0.001) compared to at 21-22 mm. In females, the decline was 1.43 mm when Rt AL was 24-25 mm, but no significant association was noted in male participants (Table 4 and Figure 2A).

**Table 4:** Association of right axial length with right corneal curvature stratified by sex. A multivariate regression model was utilized to evaluate the p-value, and the regression model was adjusted by age. mm: millimeter; β-coff: β-coefficient; 95% CI: 95% confidence interval

	Overall (N=200)		Male (n=90)		Female (n=110)	
Right axial length	β-coff (95% CI)	p-value	β-coff (95% CI)	p-value	β-coff (95% CI)	p-value
21-22 mm	Reference		Reference		Reference	
23 mm	-0.61 (-1.08, -0.13)	0.013	0.11 (-0.67, 0.89)	0.779	-0.89 (-1.52, -0.27)	0.006
24-25 mm	-1.32 (-1.84, -0.80)	<0.001	-0.79 (-1.62, 0.03)	0.058	-1.43 (-2.14, -0.72)	<0.001

A significant reduction was noted in Lt CC (β-coff=-0.65; 95% CI=-1.10, -0.20; p=0.005) when Lt AL was 23 mm, while comparing with Lt AL at 21-22 mm, the decline also remains significant for female participants (β-coff=-0.87; 95% CI=-1.48, -0.26; p=0.006), but not for male. The estimated Rt CC declined more when Rt AL was at 24-25 mm (β-coff=-1.34; 95% CI=-1.86, -0.82; p<0.001) compared to at 21-22 mm. The significant decline remains the same for males and females (p=0.012 and p<0.001, respectively) (Table 5 and Figure 2B).

**Table 5:** Association of left axial length with left corneal curvature stratified by sex. A multivariate regression model was utilized to evaluate the p-value, and the regression model was adjusted by age. mm: millimeter; β-coff: β-coefficient; 95% CI: 95% confidence interval

	Overall (N=200)		Male (n=90)		Female (n=110)	
Left axial length	β-coff (95% CI)	p-value	β-coff (95% CI)	p-value	β-coff (95% CI)	p-value
21-22 mm	Reference		Reference		Reference	
23 mm	-0.65 (-1.10, -0.20)	0.005	-0.20 (-0.87, 0.48)	0.561	-0.87 (-1.48, -0.26)	0.005
24-25 mm	-1.34 (-1.86, -0.82)	<0.001	-0.94 (-1.67, -0.21)	0.012	-1.48 (-2.24, -0.72)	<0.001

No significant association was found between Rt AL and the right axis (Table 6 and Figure 2C). Lt AL at 24-25 mm significantly increased the left axis (β-coff=16.7; 95% CI=0.81, 32.7; p=0.040) compared to axial length at 21-22 mm. No other significant association was noted (Table 7 and Figure 2D).

**Table 6:** Association of right axial length with right axis stratified by sex. A multivariate regression model was utilized to evaluate the p-value, and the regression model was adjusted by age. mm: millimeter; β-coff: β-coefficient; 95% CI: 95% confidence interval

	Overall (N=200)		Male (n=90)		Female (n=110)	
Right axial length	β-coff (95% CI)	p-value	β-coff (95% CI)	p-value	β-coff (95% CI)	p-value
21-22 mm	Reference		Reference		Reference	
23 mm	1.11 (-9.97, 12.2)	0.844	-4.10 (-21.5, 13.3)	0.641	1.69 (-13.4, 16.8)	0.825
24-25 mm	-4.35 (-16.5, 7.76)	0.480	-8.74 (-27.0, 9.54)	0.344	-4.92 (-22.0, 12.2)	0.569

**Table 7:** Association of left axial length with left axis stratified by sex. A multivariate regression model was utilized to evaluate the p-value, and the regression model was adjusted by age. mm: millimeter; β-coff: β-coefficient; 95% CI: 95% confidence interval

	Overall (N=200)		Male (n=90)		Female (n=110)	
Left axial length	β-coff (95% CI)	p-value	β-coff (95% CI)	p-value	β-coff (95% CI)	p-value
21-22 mm	Reference		Reference		Reference	
23 mm	5.51 (-8.26, 19.3)	0.431	-0.86 (-25.0, 23.2)	0.944	8.65 (-8.00, 25.3)	0.304
24-25 mm	16.7 (0.81, 32.7)	0.040	18.1 (-8.02, 44.2)	0.172	9.22 (-11.6, 30.0)	0.381

**Figure 3:**
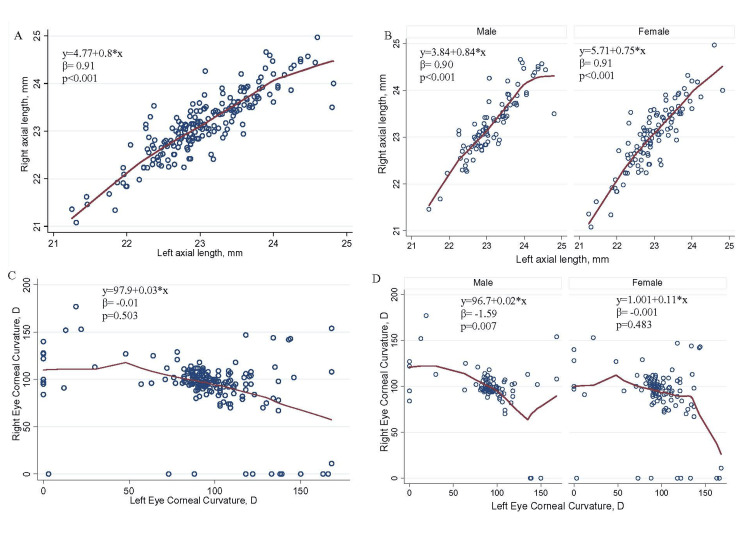
Association between Lt AL and Rt AL (A and B) and between Lt CC and Rt CC (C and D). A simple linear regression was used to see if there was a positive relationship noted between Lt AL and Rt AL and between Lt CC and Rt CC, and when stratified by male and female. Notes: Y=a+bX, where Y is the right axial length (dependent variable), a and b are unknown constants that determine the position of the line, and X is the independent variable (left axial length). β represents regression coefficient, which is the difference in the predicted (independent variable) value of the response variable (outcomes) for each one-unit change in the predictor variable, assuming all other predictor variables are held constant. Rt AL: right eye axial length; Lt AL: left eye axial length; Rt CC: right eye corneal curvature; Lt CC: left eye corneal curvature; mm: millimeter; D: diopter

There was a positive relationship noted between Lt AL and Rt AL (β-coff=0.91; 95% CI=0.84, 0.98; p<0.001). The association remained the same when stratified by male and female (Figure 3A, 3B). No significant association was found while comparing Lt CC and Rt CC, but a significantly negative association (β-coff=-1.59; 95% CI=-2.72, -0.46; p=0.007) was found in male participants while the association was checked separately in male and female (Figure 3C, 3D).

## Discussion

The computation of the AL of the eyeball and corneal curvature are well-established diagnostic aids for different clinical conditions in ophthalmology, especially in refractive error [44,45]. Globally, refractive error is the second highest cause of loss of sight and low vision [46]. Ocular biometry is the quantification of the anatomical length and breadth of the eye, which encompass corneal curvature (keratometry), axial length, and anterior chamber depth. Thereby, it is one of the most influential procedures in assessing refractive errors [13-15]. The refractive components usually are the axial length and the corneal curvature, which are interdependent [14-16]. The present study was based on optical parametric measurement and included 200 adult individuals from the northern part of Bangladesh with 6/6 visual acuity without any aid. The age range in this study was 21-52 years. Most of the participants were in the age range of 21-30 years, and only 4% were in the age group of fourth and fifth decade. This study aims to see the connection between axial length and corneal curvature, and demographic characteristics in adult emmetropic Bangladeshi people.

This study showed that the AL of both eyes was statistically significantly different between the sexes. Overall, males had AL higher than females. It has been reported that the AL of the eyeball globally is considered to be 24 mm among adults, notwithstanding sex, race, and additional anatomical mensuration [47]. Thereby, Bangladeshi people’s AL was lower than the international standard. Nevertheless, one prospective Taiwanese study revealed that there was a prolongation of AL by 10 years. The mean AL was initially 23.65±1.80 mm, and after 10 years, it was increased to 24.30±1.90 with a statistically significant difference (p=0.003) [48]. The initial value of the Taiwanese study was similar to the current study’s findings. As our study was cross-sectional, we cannot assess AL change after 10 years in the same subjects. Nonetheless, age-related prolongation of AL has been evident in other studies [49,50].

Astigmatism is a frequently found ocular disease, but this visual defect is a correctable refractive error [51]. It is due to the incompatibility between corneal or lens curvature error, leading to blurred distance and near vision [52,53]. Females of the current study participants had higher CC than their male counterparts. However, there were statistically significant differences observed only in Lt CC. Multiple studies reported that females’ CC are steeper or higher than males’ [54,55]. The current study findings were in the same line as the earlier studies.

This study found that when the AL of the eyeball increases, the CC decreases. Additionally, it has been observed that increasing AL was more statistically significantly inversely associated with decreasing CC of both the right and left eyes. Our findings were similar to studies conducted in Nigeria [5], the USA [56], and China [57]. The Nigerian study revealed that there was a statistically significant inverse correlation between AL and CC (r=-0.53; p<0.0001) [5]. Axial length, corneal curvature, and anterior chamber depth are strongly responsible for refractive error [56]. Furthermore, genetic influences also exist for anomalous measurements of the eye [56]. In multivariate analysis, AL was significantly associated with a higher corneal curvature radius (p<0.001) and negatively associated with the lens vault (p<0.001), which is equally accountable for developing refractive error [57]. Multiple studies have reported that reducing or flattening corneal curvature promotes progressive hyperopia and corneal astigmatism [58-60]. Furthermore, no statistically significant association was found between Rt AL and the right axis; nonetheless, a significant association was observed between Lt AL and the left axis. There was a statistically significant positive association found between Lt AL and Rt AL. This association remained the same when stratified between sexes. However, no significant association was found when comparing Lt CC and Rt CC. Still, a significant negative association was found in male participants while the association was observed when checked separately between the sexes.

Limitations of this research

This study sample size was small, only 200. If it was more extensive, the results would be more accurate. The sample was not equal in each group of age. Time and finances were considered constraints and significant obstacles to conducting this research more widely.

## Conclusions

It was revealed from the results of this study that there were two critical features of the eyeball, i.e., corneal curvature and axial length, which are the most important issues to maintain 6/6 visual acuity in the emmetropic eye. There is a relationship between them, which might be utilized in various applications in medical science, including ophthalmology, especially in measuring the power of the intraocular lens in cataract surgery and other ocular surgery. This significant relation also helps in the recruitment of various jobs such as military services, navy services, aviation or pilot, driver, railways, and traffic police, in which accurate vision of the individual is vital. The study’s findings might provide supplementary information to other researchers in this domain.

A further large-scale study is recommended as the present study was conducted in a limited territory. Observing axial length and corneal curvature using an improved instrument such as an optical biometer is also recommended to obtain a more accurate value.
